# Prefrontal intra-individual ERP variability and its asymmetry: exploring its biomarker potential in mild cognitive impairment

**DOI:** 10.1186/s13195-024-01452-5

**Published:** 2024-04-13

**Authors:** Joel Eyamu, Wuon-Shik Kim, Kahye Kim, Kun Ho Lee, Jaeuk U. Kim

**Affiliations:** 1 Digital Health Research Division, Korea Institute of Oriental Medicine, Daejeon, South Korea; 2 KM Convergence Science, University of Science and Technology, Daejeon, South Korea; 3 Gwangju Alzheimer’s Disease and Related Dementias (GARD) Cohort Research Center, Chosun University, Gwangju, South Korea; 4 Department of Biomedical Science, Chosun University, Gwangju, South Korea; 5 Dementia Research Group, Korea Brain Research Institute, Daegu, South Korea

**Keywords:** Mild cognitive impairment, Alzheimer’s disease, Electroencephalography, Event-related potential, Trial-to-trial variability, Intra-individual variability, Asymmetry, Screening tool

## Abstract

**Background:**

The worldwide trend of demographic aging highlights the progress made in healthcare, albeit with health challenges like Alzheimer’s Disease (AD), prevalent in individuals aged 65 and above. Its early detection at the mild cognitive impairment (MCI) stage is crucial. Event-related potentials (ERPs) obtained by averaging EEG segments responded to repeated events are vital for cognitive impairment research. Consequently, examining intra-trial ERP variability is vital for comprehending fluctuations within psychophysiological processes of interest. This study aimed to investigate cognitive deficiencies and instability in MCI using ERP variability and its asymmetry from a prefrontal two-channel EEG device.

**Methods:**

In this study, ERP variability for both target and non-target responses was examined using the response variance curve (RVC) in a sample comprising 481 participants with MCI and 1,043 age-matched healthy individuals. The participants engaged in auditory selective attention tasks. Cognitive decline was assessed using the Seoul Neuropsychological Screening Battery (SNSB) and the Mini-Mental State Examination (MMSE). The research employed various statistical methods, including independent t-tests, and univariate and multiple logistic regression analyses. These analyses were conducted to investigate group differences and explore the relationships between neuropsychological test results, ERP variability and its asymmetry measures, and the prevalence of MCI.

**Results:**

Our results showed that patients with MCI exhibited unstable cognitive processing, characterized by increased ERP variability compared to cognitively normal (CN) adults. Multiple logistic regression analyses confirmed the association between ERP variability in the target and non-target responses with MCI prevalence, independent of demographic and neuropsychological factors.

**Discussion:**

The unstable cognitive processing in the MCI group compared to the CN individuals implies abnormal neurological changes and reduced and (or) unstable attentional maintenance during cognitive processing. Consequently, utilizing ERP variability measures from a portable EEG device could serve as a valuable addition to the conventional ERP measures of latency and amplitude. This approach holds significant promise for identifying mild cognitive deficits and neural alterations in individuals with MCI.

**Supplementary Information:**

The online version contains supplementary material available at 10.1186/s13195-024-01452-5.

## Introduction

The worldwide trend of demographic aging highlights the progress made in healthcare over the last century [1]. Nonetheless, this aging population encounters diverse health issues, notably Alzheimer’s Disease (AD), pronounced in those aged 65 or older [2]. AD, the primary form of dementia (contributing 60–70% of cases) affects 55 million worldwide, with projections estimating over 150 million affected individuals by 2050 [3].

Mild Cognitive Impairment (MCI) is a syndrome marked by cognitive decline that exceeds what is typical for a person’s age and educational level, yet doesn’t significantly disrupt their daily life activities [4]. Occurring between normal cognitive function and dementia, it exhibits an annual progression rate to dementia ranging from 8 to 15% and is estimated to affect 15–20% of persons aged 60 and above [5]. The increasing prevalence of MCI presents a considerable health and economic challenge worldwide, thus the urgent necessity for its detection [6, 7]. Timely identification facilitates effective disease management, enabling prompt therapeutic intervention and the implementation of preventive healthcare measures. Such an approach can halt disease progression and alleviate the emotional and financial strains faced by patients and caregivers [8].

One method to understand neural activity and cognitive performance is the use of electroencephalography (EEG). It’s a crucial tool for exploring indicators of cognitive impairments [9, 10], primarily by analyzing event-related potentials (ERPs) - the brain’s responses to sensory stimuli or tasks involving motor and cognitive functions [11]. This allows researchers to observe the sequential unfolding of cognitive processes before sensory information reaches the peripheral nervous system, continuing after the execution of a behavioral response [12].

Various ERP components serve as indicators for sensory, attentional, and cognitive processes. One such component, the P200, occurring around 200 ms after stimulus onset, reflects exogenous sensory attention or the sensation-seeking behavior of an individual [13, 14]. Studies have shown delayed P200 latency in patients with AD [15, 16], indicating disruptions in sensory attention processes in these individuals. Conversely, the P300 component is associated with attention allocation, and engagement of working memory, and reflects novel information processing and revision of memory representations in the central nervous system (CNS) [17, 18]. Discrepancies in these ERP components, observed during basic stimulus discrimination tasks, offer valuable insights into individual differences in sensory attention capabilities [15], cognitive processing efficiency, and speed [18]. This makes them essential tools for cognitive assessment, enabling the identification and tracking of the onset and progression of neurodegenerative diseases [19].

An essential part of the conventional ERP methodology includes averaging numerous segments of EEG signals for repeated stimulus events. This presupposes that the fundamental stimulus or response-locked signal remains consistent across trials, representing an unchanging psychological process [20, 21]. Nonetheless, it is highly improbable for any psychological process of interest to be evoked in a precisely identical manner across trials due to the differences in stimulus or response properties, the effects of learning or habituation, fatigue during extended experimental tasks, or random variations in engagement. Furthermore, the process of averaging ERPs can mask important complementary functional information, such as variations in information processing observed in single-trial ERPs which could be crucial for understanding the nuances of cognitive functioning [21]. Therefore, analyzing ERP variability becomes indispensable in understanding the fluctuations within psychophysiological processes of interest.

Researchers have explored intra-individual variability through brain imaging, reaction time (RT), sensorimotor, and cognitive performances [22–24]. By analyzing fluctuations within a subject across successive trials [26], the stability of task performance can be assessed, where lower variability suggests superior performance and higher variability indicates poorer performance [22–24]. This variability is thought to reflect relatively consistent endogenous factors, such as the integrity of the CNS [27], with its increase connected to processes mediated by the frontal cortex, such as attentional lapses [28] and variations in executive control [29].

One method for analyzing variability is the response variance curve (RVC) [30]. It quantifies variance from individual trial ERPs within the average ERP, assessing data point variability within a specific timeframe. This approach addresses the challenges of analyzing single-trial variability (as used by [31]), especially when a poor signal-to-noise ratio complicates individual trial analysis [30]. Moreover, it would be appropriate in conditions that diminish neural plasticity like in late-stage MCI [32], which could contribute to a low signal-to-noise ratio [33].

This method has been utilized in distinguishing individuals with Attention Deficit and Hyperactivity Disorder (ADHD) [21] and Schizophrenia [34, 35] from those without these conditions. This distinction is based on the belief that ADHD and Schizophrenia are characterized by attention deficits linked to the variability in the CNS. To date, scanty studies have investigated ERP variability in AD/MCI and cognitively healthy elderly. Some, like [36] have used the trial-to-trial variability approach while others such as [37] have attempted to investigate variability based on the test-retest reliability of the ERPs. Patterson et al. [36] analyzed latency variability in auditory ERPs (N1, P2, N2, and P3 components) revealing that people with dementia had extended P3 latencies and increased P3 variability compared to healthy individuals.

While exploring the trial-to-trial variability, we also seek to understand if these variabilities are asymmetrical in the two prefrontal hemispheres. Right–left asymmetry of the human brain is a fundamental characteristic, albeit complex and influenced by numerous confounding factors [39], though the extent to which it varies with these factors like age, sex, handedness, brain size, and heredity, is still questionable [40]. Due to increased activity in the right prefrontal lobe and withdrawal reactions to unpleasant stimuli, frontal asymmetry is an indicator of depression [41, 42].

Measures of EEG-based asymmetries, such as Frontal Alpha Asymmetry (FAA) are linked to depression and mood studies [43], while other research endeavors have delved into auditory ERP asymmetries in sound perception [44]. Given depression’s prevalence in MCI patients [45], investigating trial-to-trial variability asymmetry could prove a potential predictive feature for MCI. Recent studies, such as [46], showcase the viability of modern low-density channel EEG devices in primary care and outpatient contexts due to their portability, cost-efficiency, and accessibility. Our prior investigations [47–50], utilizing a portable 2-channel EEG device positioned at Fp1 and Fp2 according to the 10–20 setup, consistently underscored the promising potential of portable EEG technology in the realm of AD/MCI diagnosis and assessment.

This study aimed to assess the practicality of utilizing trial-to-trial variability and its asymmetry from a portable EEG system in the detection of MCI, focusing on both non-task and task-relevant neural responses in the P200 and P300 ERP components. We hypothesized that increased ERP variability is indicative of MCI-related fundamental neurological destabilization and deficits which result in unstable cognitive processing and decreased task performance.

Primarily, this study seeks to examine: (1) if measures of ERP variability in responses to target and non-target stimuli can distinguish between elderly cognitively healthy and patients with MCI; (2) if ERP variability asymmetry can distinguish the two groups: and (3) the relationships between demographic and neuropsychological measures, ERP variability and its asymmetry measures, and the prevalence of MCI.

To the best of our knowledge, this is the only study that has investigated trial-to-trial ERP variability and its asymmetry between the MCI and cognitively healthy elderly in a relatively larger sample size using a portable EEG device.

## Materials and methods

### Participants

This study comprised 1,616 participants enlisted from October 2018 to December 2021 at the Gwangju Alzheimer’s Disease and Related Dementias (GARD) center in Gwangju City, South Korea. For analysis, 348 participants were excluded for various reasons: not falling into the CN or MCI categories [*n* = 85], had no trials with amplitudes within ± 100 µV in either target or non-target responses in any of the two channels [*n* = 14], contained less than 50% of clean trials [*n* = 64] or contained extreme variability values [*n* = 61], having incomplete neuropsychological information [*n* = 19], and lacking discernable peaks in the RVC curves [*n* = 105] (See Figure S1 in the supplementary material).

The study involved aged-matched participants categorized into two groups CN and MCI. The categorization was performed following the methodology outlined in [51], which stated, “A comprehensive clinical interview was conducted for all participants, assessing their Clinical Dementia Rating (CDR). CN participants demonstrated a CDR score of 0, indicating typical cognitive function, and exhibited no signs of brain atrophy, white matter changes, lacunae, infarction, or other focal brain lesions in their magnetic resonance imaging (MRI) scans. In contrast, participants diagnosed with MCI met the criteria outlined by [52] and showed a CDR score of 0.5. Their performance on neuropsychological tests fell below − 1.5 standard deviations on at least one of the five domain tests, adjusted for age, education, and sex-specific norms”.

For the final analysis, the CN group consisted of 878 participants, comprising 386 men and 492 women, while the MCI group comprised 390 participants, including 194 men and 196 women.

### Neuropsychological battery

The cognitive abilities of participants were assessed using the latest edition of the SNSB (SNSB II) [53, 54]. It encompasses five cognitive domains: attention, language, memory, visuospatial skills, and frontal/executive functions. Widely recognized in South Korea, this neuropsychological screening battery is a standard tool frequently utilized to evaluate cognitive function in individuals with MCI or dementia. Furthermore, the primary screening tool for cognitive assessment was the Korean version of the Mini-Mental State Examination (K-MMSE).

### ERP recording

The NeuroNicle FX2 (LAXTHA, Daejeon, South Korea) was utilized to record EEG. Based on the 10–20 system, two monopolar scalp electrodes were placed at Fp1 and Fp2, utilizing the right earlobe as the reference. A bandstop filter ranging from 55 to 65 Hz was utilized, and the contact impedances for all EEG electrodes were kept below 10 kΩ. With a 250 Hz sampling frequency and a 15-bit resolution, data were digitized in continuous recording mode.

During recording, participants were instructed to sit comfortably with their eyes closed, while qualified operators monitored both participant sleepiness and EEG traces to minimize artifacts from muscle and eye movements. ERPs were elicited using an active auditory oddball task, where 64 rare random target stimuli at 2,000 Hz (1/5 ratio) and 256 standard auditory stimuli at 750 Hz (4/5 ratio) were presented [47, 48, 55]. The EEG signals were acquired in a sequence of conditions of 5 min of resting state, 8 min of sensory-evoked potentials, and 5 min of selective attention tasks. More on the EEG acquisition procedure is described in our previous study [47].

In this study, the focus was solely on selective attentional ERPs. Before the experiment, participants were evaluated for auditory hearing capabilities for the rare (2,000 Hz) and standard (750 Hz) tones. Moreover, their ability to discern between the tones was assessed using earphones with a volume level fixed at 70 dB. The participants were further told to press a response key upon identifying a rare tone. To maintain a controlled environment during data collection, all the experiments were conducted in a quiet room with standardized illumination.

### EEG data processing

Tailored Python scripts *(version 3.8.16)* were used to process the data. The EEG data underwent the following preprocessing procedures to extract the artifact-free trials; data segmentation [− 200, 800] ms (with 0 ms as the stimulus onset), baseline correction [− 200, 0] ms, artifact removal [± 100 µV as the threshold], and the final ERP traces were subjected to a nine-order moving average filter. These procedures were applied specifically to correct trials (standard stimuli that received no response and target stimuli that were accurately identified).

To consolidate the data, extracted trial-to-trial variability measures from the prefrontal channels Fp1 and Fp2 were averaged, creating a unified dataset for subsequent analysis. Furthermore, participants who didn’t meet the minimum threshold of 50% of clean trials in the standard and odd neural responses in the two channels were not considered for further analysis.

### Response variance curve (RVC) measures

**Fig. 1:**
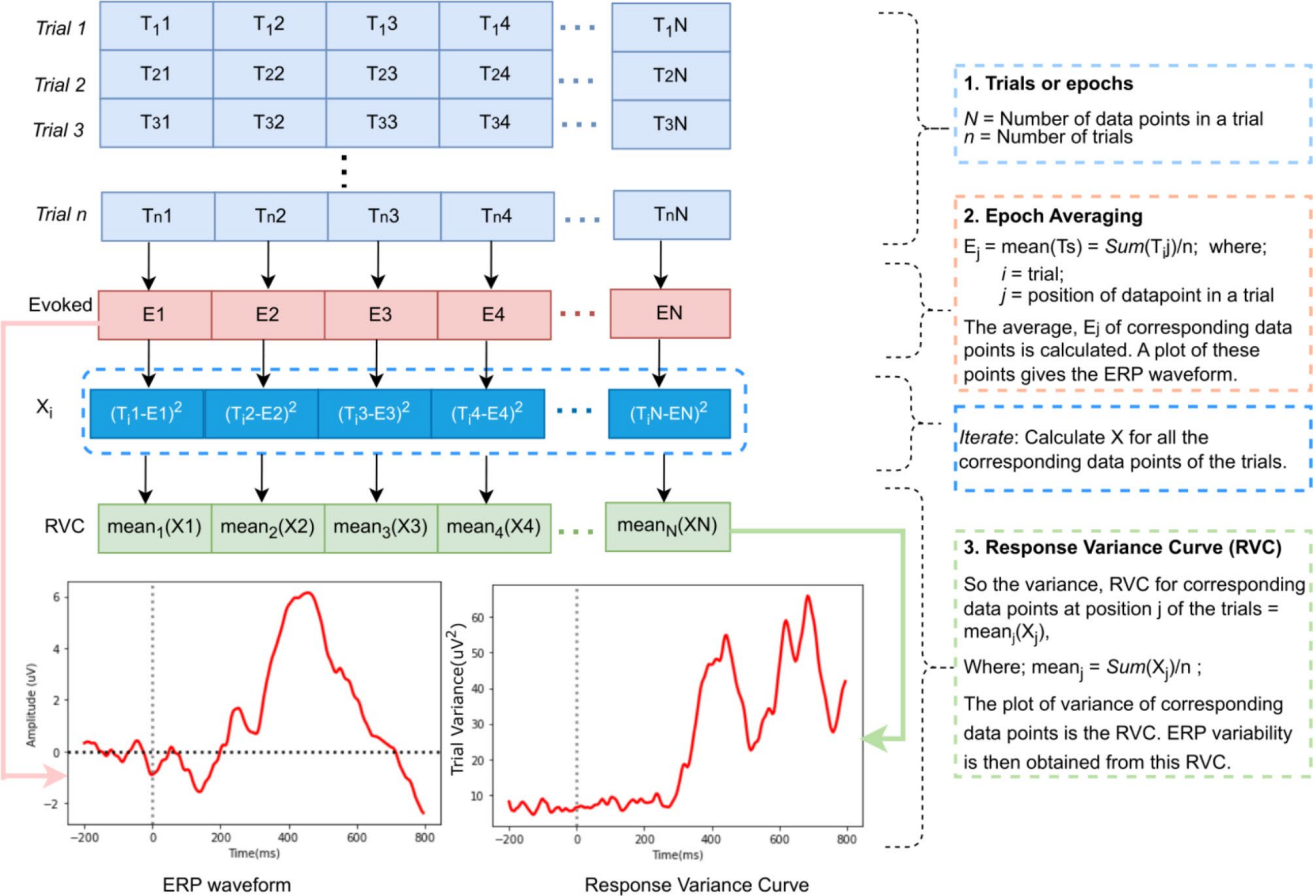
The ERP and RVC derivation process

First, we derived the RVC [30, 56] (See Fig. 1) and obtained the ERP variability measures of the P200 and P300 ERP components in two respective ERP windows of 150–300 ms and 300–600 ms after stimulus onset for both the target and non-target neural responses.

The RVC measures of variabilities in the Peak Amplitude (AMPV), Latency (LATV), 50% Fractional Area Latency (FALV), and the Area Under the Curve (AUCV) were extracted.

### RVC asymmetry measures

From the variability measures extracted above, we derived corresponding normalized asymmetry measures for each of the RVC measures of AMPV, AUCV, LATV, and FALV based on (Fp2-Fp1)/(FP2 + Fp1). We divided by the sum to minimize the consequences for the degree of negativity of asymmetry in both groups [57]. We thus, obtained asymmetry measures of AMPV_Asym, AUCV_Asym, LATV_Asym, and FALV_Asym (See Table S1 in supplementary for the asymmetry formula and detailed description).

### Statistical analysis

The statistical analyses were performed using *R Studio (version 2022.07.2 + 576)*, running on *R (version 4.1.3)* for Windows. The analysis included the use of packages such as *gtsummary (version 1.6.1)*, *ggplot2 (version 3.4.0)*, and *corrplot (version 0.92)* [58–61]. The significance level of *α* for all tests was set at 0.05. Independent sample t-tests were conducted using Student’s t-test and Welch’s t-test for continuous variables, while chi-squared tests were employed for categorical variables. Univariate and multiple logistic regression analyses were conducted to compute the odds ratios associated with MCI for each ERP variability and asymmetry measure. Covariates such as age, sex, and education level were controlled for in the analyses. The MMSE score was included as an additional covariate to evaluate the independent relationship between ERP variability, the corresponding asymmetry measures, and MCI.

## Results

### Participant characteristics

Table 1, consists of demographic and neuropsychological measures of the participants under consideration for analysis. The study included 390 patients with MCI and 878 CN. The patients with MCI had 50% women and 50% men while the CN group had 56% women and 44% men. Patients with MCI were older than CN individuals, with mean age ± standard deviation of 73.82 ± 6.54 and 72.06 ± 6.36 years (*p* < 0.001) respectively. Expectedly, patients with MCI had lower MMSE scores than CN, with 26.31 ± 2.68 and 27.68 ± 1.90 scores (*p* < 0.001) respectively. Furthermore, patients with MCI had lower scores in all the SNSB II domains; attention [8.54 ± 1.94], language [− 0.09 ± 0.47], visuospatial [0.25 ± 1.89], memory [− 0.44 ± 0.63], and frontal [− 0.24 ± 0.70] compared to CN individuals [9.71 ± 2.19, 0.21 ± 0.28, 0.54 ± 0.37, 0.30 ± 0.58, and 0.23 ± 0.57] (*p* < 0.001) respectively. No statistically significant differences were observed in the sex and years of education between the two groups.

**Table 1:** Demographic characteristics and neuropsychological test domain scores

Characteristic	CN, *N* = 878^1^	MCI, *N* = 390^1^	T- statistic	p-value^2^
**Demographic characteristics**				
**Age**	72.06 (6.36)	73.82 (6.54)	-4.509	**< 0.001**
**Sex**			3.635	0.057
Female	492 / 878 (56%)	196 / 390 (50%)		
Male	386 / 878 (44%)	194 / 390 (50%)		
**EDUYR**	10.83 (4.36)	11.14 (4.48)	-1.151	0.3
**Neuropsychological test scores**				
**MMSE**	27.68 (1.90)	26.31 (2.68)	9.150	**< 0.001**
**Attention**	9.71 (2.19)	8.54 (1.94)	9.479	**< 0.001**
**Language**	0.21 (0.28)	-0.09 (0.47)	11.501	**< 0.001**
**Visuospatial**	0.54 (0.37)	0.25 (1.89)	2.960	**0.003**
**Memory**	0.30 (0.58)	-0.44 (0.63)	19.753	**< 0.001**
**Frontal**	0.23 (0.57)	-0.24 (0.70)	11.696	**< 0.001**

^1^Mean (SD); n / N (%); ^2^two sample t-test; Pearson’s Chi-squared test; Welch Two Sample t-test

Significant features (p-value ≤ 0.05) are bolded

### P2 measures

#### Standard responses

**Fig. 2:**
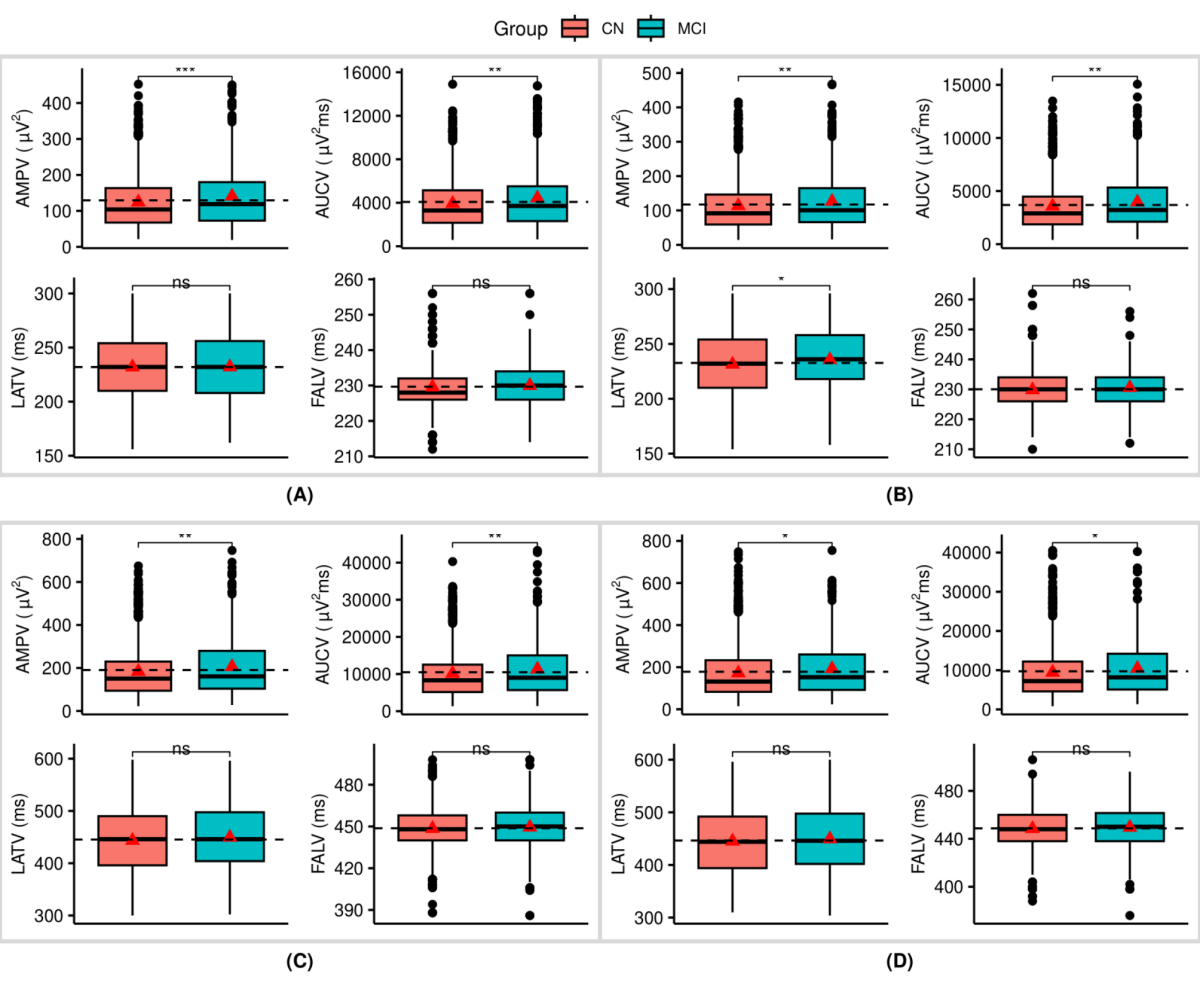
Boxplots showing group differences in the trial-to-trial variability measures based on t-tests: (**A**) P200 non-target (standard) neural responses; (**B**) P200 target(oddball) neural responses; (**C**) P300 non-target (standard) neural responses; (**D**) P300 target (oddball) neural responses; The dotted black horizontal line is the overall mean and the red triangles are the group means; Significance levels are denoted as follows: *** for *p* < 0.001, ** for *p* < 0.01, * for *p* ≤ 0.05, and ns for not significant; Detailed statistical values can be found in Table S2 of the supplementary material

Patients with MCI exhibited significantly higher variability in amplitudes and AUC values in the standard responses. Specifically, larger measures of AMPV [*t* = − 3.14, *p* = 0.002] and AUCV [*t* = − 3.08, *p* = 0.002] were observed in the MCI when compared to CN individuals. Nonetheless, no significant differences were observed in the latency variability measures for LATV and FALV among participants in either group (Fig. 2(A) and supplementary Table S2).

#### Target responses

As observed in the standard neural responses, patients with MCI exhibited significantly higher variability in amplitudes and AUC in the target neural responses i.e., AMPV [*t* = − 2.60, *p* = 0.009], and AUCV [*t* = − 2.61, *p* = 0.009] respectively, compared to CN individuals. They also had a higher LATV [*t* = − 2.35, *p* = 0.019]. Nonetheless, no significant differences were observed in the latency variability measure of FALV among participants in either group (Fig. 2(B)).

### P3 measures

#### Standard responses

Like the P2 measures, patients with MCI exhibited significantly higher variability in amplitudes and AUC values for the non-target neural responses. That is, larger AMPV [*t* = − 2.68, *p* = 0.008] and AUCV [*t* = − 2.60, *p* = 0.009] were observed when compared to CN individuals. Nonetheless, no significant differences were observed in the latency variability measures for LATV and FALV among participants in either group (Fig. 2(C)).

#### Target responses

In the target neural responses, patients with MCI exhibited significantly higher variability in amplitudes and AUC values; AMPV [*t* = − 2.55, *p* = 0.011] and AUCV [*t* = − 2.36, *p* = 0.018], when compared to CN individuals. Nonetheless, no significant differences were observed in the latency variability measures for LATV and FALV among participants in either group (Fig. 2(D)).

### P3 asymmetry measures

**Fig. 3:**
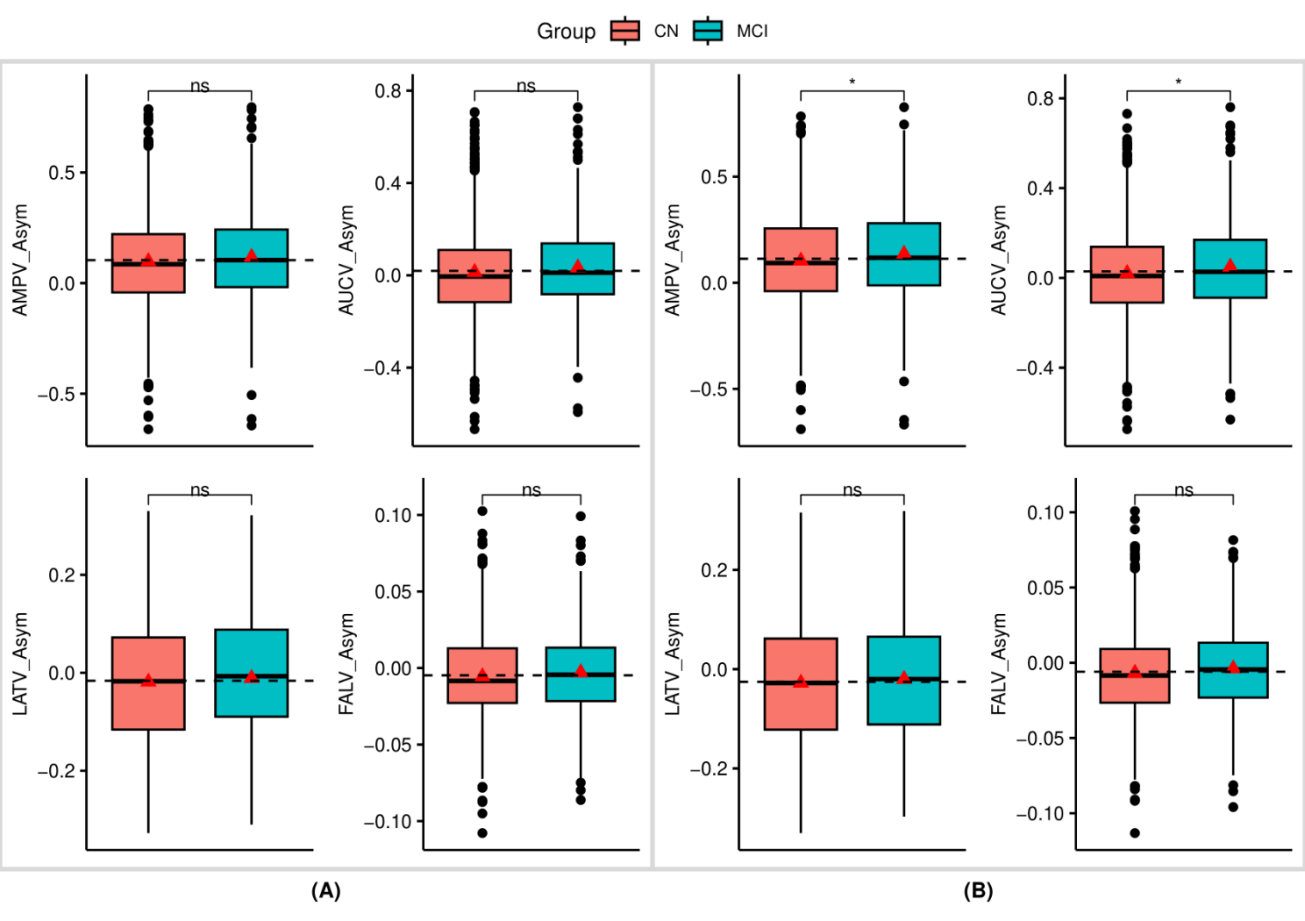
Boxplots showing group differences based on t-tests in the asymmetry measures of trial-to-trial variability : (**A**) P300 non-target neural responses; (**B**) P300 target neural responses; Significance levels are denoted as follows: *** for *p* < 0.001, ** for *p* < 0.01, * for *p* ≤ 0.05, and ns for not significant; Detailed statistical values can be found in Table S2 of the supplementary material

Patients with MCI exhibited significantly higher asymmetry in the variability of amplitudes and AUC values in the target responses. Specifically, larger measures of AMPV_Asym [*t* = − 2.32, *p* = 0.021] and AUCV_Asym [*t* = − 2.24, *p* = 0.025] were observed in the MCI when compared to CN individuals. Nonetheless, no notable differences were observed in the latency asymmetry variability measures of FALV_Asym and LATV_Asym among participants in either group (Fig. 3(B)).

## Logistic regression models

### P2 measures

#### Standard responses

In the crude or unadjusted model, specific variables showed odds ratios and corresponding 95% confidence intervals that were significantly different from 1, suggesting a possible connection with the risk of MCI. Specifically, the AMPV [OR = 1.22, *p* = 0.001] and AUCV [OR = 1.21, *p* = 0.001] demonstrated this potential association. However, measures of latency variability, LATV, and FALV did not demonstrate any significant connection with the risk of MCI.

Upon consideration of demographic characteristics such as sex, age, and education level, AMPV [OR = 1.22, *p* < 0.001] and AUCV [OR = 1.23, *p* < 0.001] persisted as MCI predictors, thus underscoring their independence from demographic characteristics in predicting MCI.

**Fig. 4:**
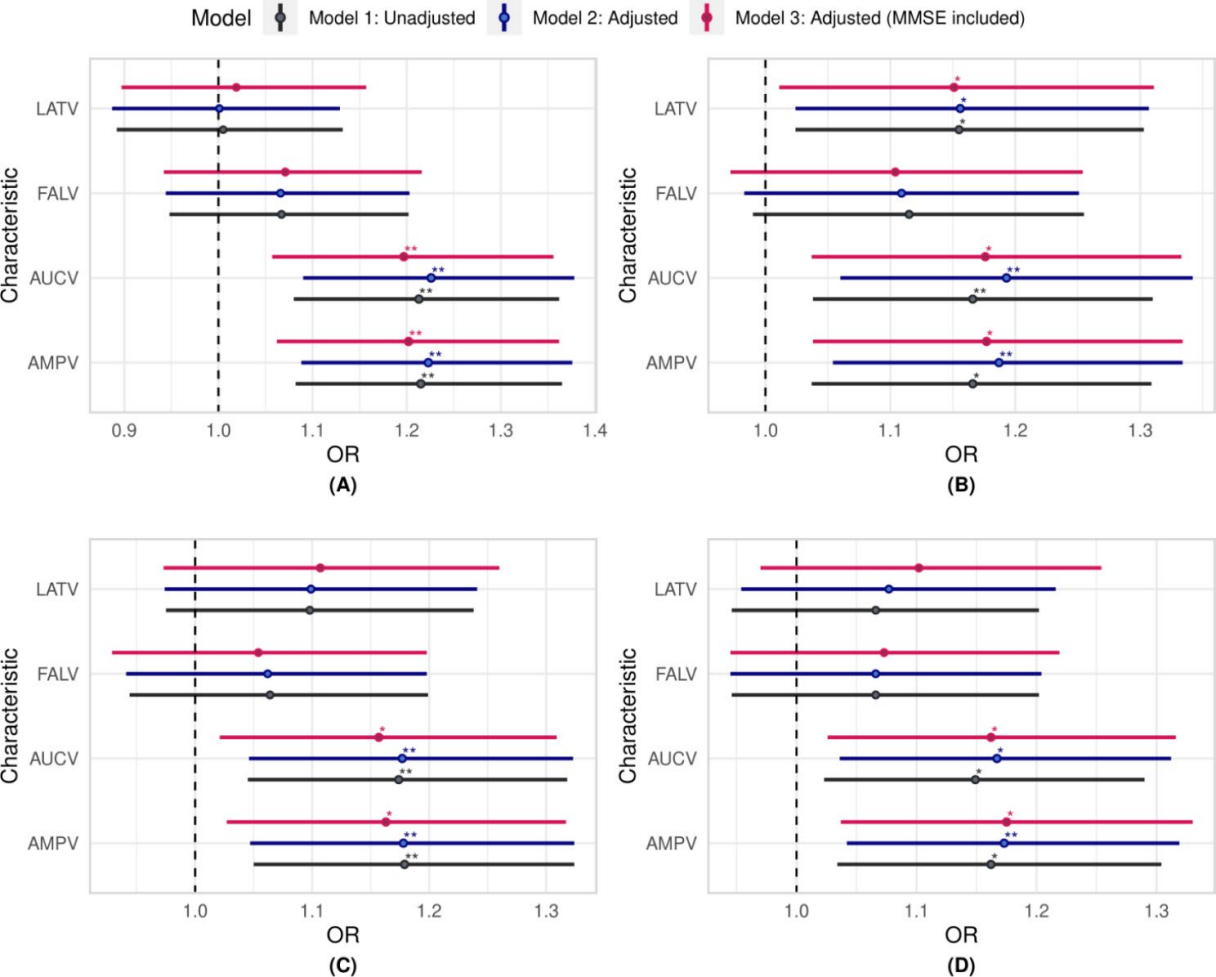
The odd ratios (OR) for the Logitistic regression (LR) models for the various trial-to-trial variability measures: (**A**) P200 non-target neural responses; (**B**) P200 target neural responses; (**C**) P300 non-target neural responses; (**D**) P3 target neural responses; Model 1 is unadjusted; Model 2 was adjusted for demographic characteristics and Model 3 was adjusted with MMSE score included; Significance levels are denoted as follows: *** for *p* < 0.001, ** for *p* < 0.01, * for *p* ≤ 0.05, and blank (no stars) for not significant; Actual p-values, Confidence Intervals (CI) and OR can be found in Table S3 of the supplementary material

On further refinement by incorporating MMSE scores, AMPV [OR = 1.20, *p* = 0.004] and AUCV [OR = 1.20, *p* = 0.005] continued to be predictors for MCI. Specifically, a 1 µV^2^ increase in AMPV and a 1 µV^2^ms increase in AUCV augmented the risk of MCI by 20%. These findings affirmed the genuine independence of these variability measures of the P200 ERP component from both demographic and MMSE measures as reliable MCI predictors (Fig. 4(A)).

#### Target responses

In the crude model, specific variables showed odds ratios and corresponding 95% confidence intervals that were significantly different from 1, suggesting a possible connection with the risk of MCI. Specifically, the AMPV [OR = 1.17, *p* = 0.010], LATV [OR = 1.15, *p* = 0.019], and AUCV [OR = 1.17, *p* = 0.010] demonstrated this potential connection. Upon incorporating demographic characteristics such as sex, age, and education level, the AMPV [OR = 1.19, *p* = 0.005], LATV [OR = 1.16, *p* = 0.019], and AUCV [OR = 1.19, *p* = 0.004] remained MCI predictors. On further refinement by incorporating MMSE scores, AMPV [OR = 1.18, *p* = 0.011], LATV [OR = 1.15, *p* = 0.033], and AUCV [OR = 1.18, *p* = 0.012] persisted as MCI predictors. A 1 µV^2^ increase in AMPV and a 1 µV^2^ms increase in AUCV augmented the risk of MCI by 18%, while a 1 ms increase in LATV augmented the risk of MCI by 15% (Fig. 4(B)).

### P3 measures

#### Standard responses

In the crude model, specific variables showed odds ratios and corresponding 95% confidence intervals that were significantly different from 1, suggesting a possible connection with the risk of MCI. Specifically, AMPV [OR = 1.18, *p* = 0.006] and AUCV [OR = 1.17, *p* = 0.007] demonstrated this potential association. However, latency variability measures of LATV and FALV had no significant connection with the risk of MCI.

Upon accounting for demographic factors such as sex, age, and education level, AMPV [OR = 1.18, *p* = 0.007] and AUCV [OR = 1.18, *p* = 0.007] persisted as MCI predictors, thus underscoring their independence from demographic characteristics in MCI prediction.

On further refinement by incorporating MMSE scores, AMPV [OR = 1.18, *p* = 0.011] and AUCV [OR = 1.16, *p* = 0.018] persisted as MCI predictors. These findings affirmed the genuine independence of these variability measures of the standard responses of the P300 ERP component from both demographic and MMSE measures as reliable MCI predictors (Fig. 4(C)).

#### Target responses

In the crude model, specific variables showed odds ratios and corresponding 95% confidence intervals that were significantly different from 1 suggesting a possible connection with the risk of MCI. Specifically, AMPV [OR = 1.16, *p* = 0.012], and AUCV [OR = 1.15, *p* = 0.020] demonstrated this connection. However, latency variability measures of LATV and FALV did not demonstrate any significant association with the risk of MCI. Upon consideration of demographic characteristics such as sex, age, and education level, AMPV [OR = 1.17, *p* = 0.008], and AUCV [OR = 1.17, *p* = 0.011] persisted as MCI predictors, thus underscoring their independence from demographic characteristics in MCI prediction.

Further adjustment by incorporating MMSE scores, AMPV [OR = 1.18, *p* = 0.011], and AUCV [OR = 1.16, *p* = 0.018] persisted as MCI predictors. These findings affirmed the genuine independence of these variability measures of the P300 ERP component from both demographic and MMSE measures as reliable MCI predictors (Fig. 4(D)).

#### P3 asymmetry measures

In the crude model, specific variables showed odds ratios and corresponding 95% confidence intervals that were significantly different from 1, suggesting a possible connection with the risk of MCI. Specifically, AMPV_Asym [OR = 1.15, *p* = 0.021], and AUCV_Asym [OR = 1.15, *p* = 0.025] demonstrated this connection. However, LATV_Asym and FALV_Asym did not demonstrate any significant association with the risk of MCI.

Upon incorporating demographic factors such as sex, age, and education level, AMPV_Asym [OR = 1.15, *p* = 0.021], and AUCV_Asym [OR = 1.15, *p* = 0.024] persisted as MCI predictors, thus underscoring their independence from demographic characteristics in MCI prediction.

**Fig. 5:**
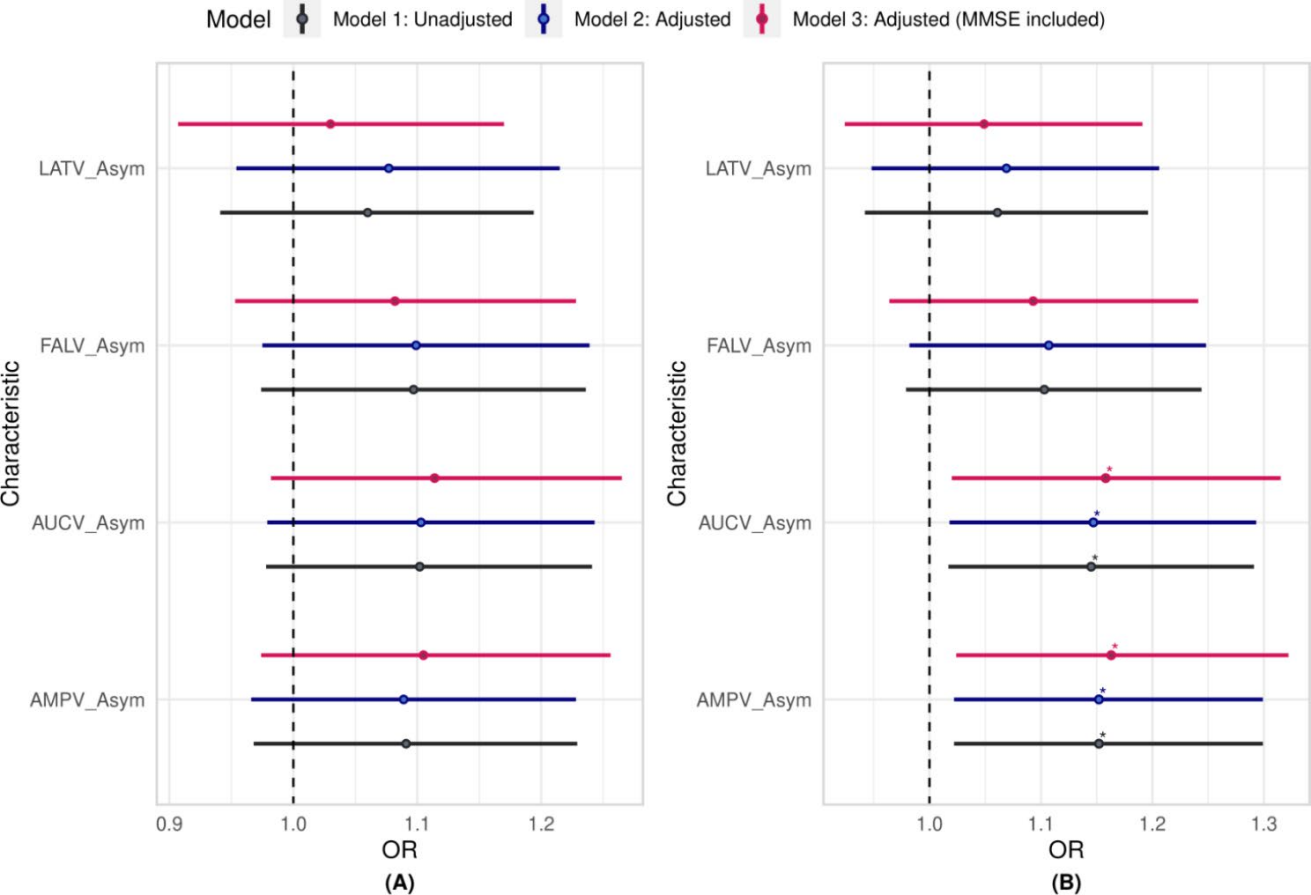
The OR for the LR models for the various variability asymmetry measures: (**A**) P300 non-target neural responses; (**B**) P300 target neural responses; Model 1 is unadjusted; Model 2 was adjusted for demographic characteristics and Model 3 was adjusted with MMSE score included; Significance levels are denoted as follows: *** for *p* < 0.001, ** for *p* < 0.01, * for *p* ≤ 0.05, and blank (no stars) for not significant; Actual p-values, CI and OR can be found in Table S3 of the supplementary materials

Further adjustment by incorporating MMSE scores revealed that AMPV_Asym [OR = 1.16, *p* = 0.020], and AUCV_Asym [OR = 1.16, *p* = 0.024] persisted as predictors for MCI. These findings affirmed the genuine independence of AMPV_Asym and AUCV_Asym from demographic and MMSE measures as reliable MCI predictors (Fig. 5(B)).

## Discussion

This study aimed to assess the potential of utilizing ERP variability and related asymmetry measures from a portable EEG system to detect MCI. Through the analysis of ERP variability and its asymmetry from an auditory oddball paradigm, a comparison was made between individuals with MCI and CN. Furthermore, the research delved into understanding the link between ERP variability plus its asymmetry and MCI prevalence taking into account the demographic and neuropsychological scores.

The trial-to-trial variability analysis revealed distinct patterns in MCI patients compared to CN individuals. The patients with MCI exhibited unstable neural activity, i.e., increased variability measures of AMPV and AUCV in standard and target responses, and had more variability asymmetry compared to the CN. This association with MCI persisted even after incorporating demographic characteristics such as age, sex, and education level, and subsequently the MMSE score. This suggests that they could potentially serve as reliable predictors for MCI, irrespective of demographic and neuropsychological measures.

First, in the analysis of the variability measures of ERP components, as hypothesized, we observed that patients with MCI had more variability compared to the CN individuals, i.e., more variability in the amplitudes (AMPV) and area under the curve (AUCV). This neural variability was observed during perceptual processing (P2) and memory updating processes (P3) in both the non-target and target neural responses. The amplitude is known to provide insights into the level of cognitive processing during a task [62] and its variability in a specified period could be an indicator of the underlying deficit in attention associated with neurogenerative diseases [63]. Amplitude also correlates with attention, stimulus identification, and memory [64], and its variability could reflect the efficiency and stability of neural networks necessary for reliable encoding and retrieval of memories [38] and so our results reveal instability and inefficiency in neural processing in the patients with MCI.

The ERP variability measures were derived from the response variance curve by assessing the variance originating from trial-to-trial ERPs utilized in the conventional average ERP [30]. This approach evaluates the variability of data points within a specific time frame, providing insights into the fluctuations in neural responses, and has been particularly useful for distinguishing conditions characterized by attention deficits linked to variability in the CNS. In the research focused on ADHD [21] and Schizophrenia [34], it was observed that patients with these conditions displayed higher variability in amplitudes when compared to individuals without these disorders. This variation was attributed to the attention deficits that are typical of both ADHD and Schizophrenia. Despite scanty studies on the use of trial-to-trial ERP variability for AD or MCI distinction from normal individuals, our study agrees with [36, 38] who reported increased ERP variability in participants with neurodegenerative diseases compared to healthy individuals. These results reveal unstable perceptual and cognitive processing in the patients with MCI compared to cognitively healthy individuals. These could be attributed to several factors: First, there could be variability in the severity and progression of cognitive decline in patients with MCI due to their heterogeneity [52, 65], thereby influencing heightened disparities observed in ERP patterns in these individuals. Additionally, due to the larger proportion of older individuals with MCI in our dataset (mean age approximately 74 years), we could attribute the heightened neural variability in MCI to diminished neural plasticity, a characteristic feature of late-stage MCI [32]. Moreover, the increased variability could potentially have maladaptive effects on perception, likely stemming from increased internal noise [33]. After accounting for demographic factors such as age, sex, and education level, as well as the MMSE score, the neural variability measures showed a notable association with MCI. This implies that they may serve as dependable predictors for MCI, regardless of demographic and neuropsychological factors.

Next, in the analysis of neural variability asymmetry, we observed heightened measures in the MCI compared to the CN. Because, a greater peak amplitude or AUC of P3 ERP corresponds to more neural activity, a positive asymmetry corresponds to more activity in the right hemisphere while a negative asymmetry measure corresponds to more activity in the left hemisphere. Extending this interpretation to the asymmetry result indicates that there was more neural variability in the right hemisphere compared to the left hemisphere in both groups, with it being pronounced in the MCI compared to the CN group. The greater ERP variability in the right hemisphere compared to the left hemisphere in both groups and generally in MCI compared to the CN may not be surprising as there is a reported prevalence of depression in patients with MCI of approximately 32% [45], with depression linked to increased activity in the right prefrontal lobe and withdrawal reactions to unpleasant stimuli, frontal asymmetry [41, 42]. According to [66] the asymmetries observed in late ERP components, such as the P300, are likely contingent upon the nature of cognitive processing and the dominant hemisphere of the participant, underscoring that these asymmetries are more connected to asymmetrical cognitive processes than to fixed structural distinctions in the hemispheres. Studies like [43, 66] have explored asymmetries based on the EEG/ERP, however, to the best of our knowledge we are not aware of studies that have explored asymmetry of trial-to-trial variability. The corresponding asymmetry measures of neural variability displayed a significant association with MCI, even after adjusting for demographic factors such as age, sex, and education level, as well as the MMSE score. This reaffirms their potential as reliable predictors for MCI, independent of demographic and neuropsychological factors.

Therefore, trial-to-trial variability and its associated asymmetry measures can be useful markers differentiating MCI, independent of neuropsychological screening tests like the MMSE, and are promising complements to the MMSE. They could also serve as valuable complements to the widely used ERP amplitude and latency measures. We hope that these features can be leveraged to improve EEG/ERP-based models, similar to those discussed in studies [48, 67–71], for screening MCI/AD.

This study had some limitations. Firstly, variability in neural responses could be due to some factors for instance the effects of learning or habituation, fatigue during extended experimental tasks, or random variations in engagement, etc., which were uncontrollable factors in our study design. Secondly, it’s important to note that our findings might have limited generalizability since our data is composed of exclusively ethnically Korean participants. Thirdly, within the group of MCI participants, there was a mix of phenotypes (amnestic and non-amnestic) with unaccounted heterogeneity within these phenotypes. This lack of distinction might impact the interpretation of our results. Finally, it’s worth noting that the discontinuity of the ERPs may have been caused by the elimination of noisy epochs, as described in our methodology. Regrettably, this aspect was not accounted for in our analysis and interpretation of results.

In conclusion, this study showcased the potential utility of intra-individual ERP variability and its associated asymmetry measures obtained from a portable EEG device in differentiating MCI from CN elderly. We extensively described these variability and asymmetry measures and explored their connection with MCI. Our results indicated that individuals with MCI exhibited increased ERP variability and higher variability asymmetry compared to CN individuals. The logistic regression analysis unveiled that ERP variability and its related asymmetry measures retained statistical significance despite adjusting for demographic and neuropsychological measures. This suggests that ERP variability could not only complement traditional neuropsychological tests but also enhance conventional ERP measures of amplitude and latency in screening mild cognitive deficits. To the best of our knowledge, no prior studies have investigated trial-to-trial variability in MCI, especially using a relatively larger sample size.

In future research endeavors, it would be crucial to substantiate these results by expanding the study to include a diverse ethnic population given that the current investigation was limited to a Korean population. Moreover, investigating these findings by focusing on a relatively homogeneous MCI subgroup, such as amnestic MCI patients, would help mitigate the influence of MCI patient heterogeneity on the present results. Furthermore, to enhance predictive models for MCI, it is essential to consider integrating these ERP variability measures alongside conventional ERP measures and/or neuropsychological test scores. This comprehensive approach could potentially lead to more accurate and reliable assessments of cognitive deficits and improve our understanding of related neurobiological processes.

### Electronic supplementary material

Below is the link to the electronic supplementary material.

Supplementary Material 1

## Data Availability

The datasets used and/or analyzed during the current study are available from the corresponding author on reasonable request.

## References

[1] International AD. Alzheimer’s Disease International. Dementia statistics. https://www.alzint.org/about/dementia-facts-figures/dementia-statistics/.

[2] Report S, Patient THE, In J, Era AN, New OF. 2023 Alzheimer’s disease facts and figures. Alzheimer’s and Dementia. 2023;19(4):1598–695. 10.1002/alz.13016 36918389

[3] Nichols E, Steinmetz JD, Vollset SE, Fukutaki K, Chalek J, Abd-Allah F (2022). Estimation of the global prevalence of dementia in 2019 and forecasted prevalence in 2050: an analysis for the global burden of Disease Study 2019. Lancet Public Health.

[4] Gauthier S, Reisberg B, Zaudig M, Petersen CR, Ritchie K, Broich K. Mild cognitive impairment. International Psychogeriatric Association Expert Conference on mild cognitive impairment. 2006.

[5] Petersen RC. Mild cognitive impairment. CONTINUUM Lifelong Learning in Neurology [Internet]. 2016;22(2, Dementia):404–18. Available from: www.ContinuumJournal.com. 10.1212/CON.0000000000000313 PMC5390929 27042901

[6] Dubois B, Feldman HH, Jacova C, DeKosky ST, Barberger-Gateau P, Cummings J (2007). Research criteria for the diagnosis of Alzheimer’s disease: revising the NINCDS-ADRDA criteria. Lancet Neurol.

[7] Mantzavinos V, Alexiou A (2017). Biomarkers for Alzheimer’s Disease diagnosis. Curr Alzheimer Res.

[8] Missonnier P, Gold G, Fazio-Costa L, Michel JP, Mulligan R, Michon A (2005). Early event-related potential changes during working memory activation predict rapid decline in mild cognitive impairment. Journals Gerontol - Ser Biol Sci Med Sci.

[9] Lai CL, Lin RT, Liou LM, Liu CK. The role of event-related potentials in cognitive decline in Alzheimer’s disease. Clinical Neurophysiology [Internet]. 2010;121(2):194–9. 10.1016/j.clinph.2009.11.001. 10.1016/j.clinph.2009.11.001 20005164

[10] Nessler D, Friedman D, Johnson R, Bersick M. ERPs suggest that age affects cognitive control but not response conflict detection. Neurobiol Aging [Internet]. 2007;28(11):1769–82. https://linkinghub.elsevier.com/retrieve/pii/S0197458006002685. 10.1016/j.neurobiolaging.2006.07.011 16930775

[11] Kappenman ES, Luck SJ. ERP Components: The Ups and Downs of Brainwave Recordings. In: Kappenman ES, Luck SJ, editors. The Oxford Handbook of Event-Related Potential Components [Internet]. Oxford Handbooks Online; 2012. pp. 1–29. https://academic.oup.com/edited-volume/34558/chapter/293240277.

[12] Woodman GF (2010). A brief introduction to the use of event-related potentials in studies of perception and attention. Atten Percept Psychophys.

[13] Katada E, Sato K, Ojika K, Ueda R. Cognitive event-related potentials: useful clinical information in Alzheimer’s Disease. 1, Curr Alzheimer Res. 2004. 10.2174/1567205043480609 15975087

[14] Sur S, Sinha VK (2009). Event related pontetial: an overview. Industral Psychiatry J.

[15] Martinelli V, Locatelli T, Comi G, Lia C, Alberoni M, Bressi S (1996). Pattern visual evoked potential mapping in Alzheimer’s disease: correlations with visuospatial impairment. Dementia.

[16] Missonnier P, Deiber MP, Gold G, Herrmann FR, Millet P, Michon A et al. Working memory load–related electroencephalographic parameters can differentiate progressive from stable mild cognitive impairment. Neuroscience [Internet]. 2007;150(2):346–56. https://linkinghub.elsevier.com/retrieve/pii/S0306452207011116. 10.1016/j.neuroscience.2007.09.009 17996378

[17] Polich (2007). Updating P300: an integrative theory of P3a and P3b. Clin Neurophysiol.

[18] Polich J, Kok A. Cognitive and biological determinants of P300: an integrative review. Biol Psychol [Internet]. 1995;41(2):103–46. https://rccardiologia.com/previos/RCC 2014 Vol. 21/RCC_2014_21_5_SEP-OCT/RCC_2014_21_5_275.pdf. 10.1016/0301-0511(95)05130-9 8534788

[19] Medvidovic S, Titlic M, Maras-Simunic M (2013). P300 evoked potential in patients with mild cognitive impairment. Acta Informatica Med.

[20] Volpert-esmond HI. Looking at Change : Examining Meaningful Variability in Psychophysiological Measurements. Biol Psychiatry Cogn Neurosci Neuroimaging [Internet]. 2022;7(6):530–1. 10.1016/j.bpsc.2022.02.006. 10.1016/j.bpsc.2022.02.006 35680343

[21] Lazzaro I, Anderson J, Gordon E, Clarke S, Leong J, Meares R. Single trial variability within the P300 (250–500 ms) processing window in adolescents with attention deficit hyperactivity disorder. Psychiatry Res [Internet]. 1997;73(1–2):91–101. https://linkinghub.elsevier.com/retrieve/pii/S0165178197001078. 10.1016/s0165-1781(97)00107-8 9463842

[22] Nesselroade JR, Salthouse TA (2004). Methodological and theoretical implications of Intraindividual Variability in Perceptual-Motor Performance. Journals Gerontol - Ser B Psychol Sci Social Sci.

[23] Naik S, Adibpour P, Dubois J, Dehaene-Lambertz G, Battaglia D. Event-related variability is modulated by task and development. NeuroImage. 2023;276(March). 10.1016/j.neuroimage.2023.120208 37268095

[24] Phillips M, Rogers P, Haworth J, Bayer A, Tales A. Intra-individual reaction time variability in mild cognitive impairment and Alzheimer ’ s Disease : gender, Processing load and speed factors. 2013;8(6). 10.1371/journal.pone.0065712 PMC3677873 23762413

[25] Li F, Wang G, Jiang L, Yao D, Xu P, Ma X et al. Disease-specific resting-state EEG network variations in schizophrenia revealed by the contrastive machine learning. Brain Res Bull [Internet]. 2023;202(June):110744. https://linkinghub.elsevier.com/retrieve/pii/S0361923023001697. 10.1016/j.brainresbull.2023.110744 37591404

[26] Wojtowicz M, Berrigan LI, Fisk JD (2012). Intra-individual variability as a measure of information processing difficulties in multiple sclerosis. Int J MS Care.

[27] MacDonald SWS, Nyberg L, Bäckman L (2006). Intra-individual variability in behavior: links to brain structure, neurotransmission and neuronal activity. Trends Neurosci.

[28] Bunce DJ, Warr PB, Cochrane T. Blocks in choice responding as a function of age and physical fitness. Psychol Aging [Internet]. 1993;8(1):26–33. http://doi.apa.org/getdoi.cfm?doi=10.1037/0882-7974.8.1.26. 10.1037//0882-7974.8.1.26 8461111

[29] West R, Murphy KJ, Armilio ML, Craik FIM, Stuss DT (2002). Lapses of intention and performance variability reveal age-related increases in fluctuations of executive control. Brain Cogn.

[30] Anderson J, Rennie C, Gordon E, Howson A, Meares R (1991). Measurement of maximum variability within event related potentials in schizophrenia. Psychiatry Res.

[31] Michalewski HJ, Prasher DK, Starr A. Latency variability and temporal interrelationships of the auditory event-related potentials (N1, P2, N2, and P3) in normal subjects. 1986. 10.1016/0168-5597(86)90037-7 2416547

[32] Clément F, Gauthier S, Belleville S (2013). Executive functions in mild cognitive impairment: emergence and breakdown of neural plasticity. Cortex.

[33] Dinstein I, Heeger DJ, Behrmann M. Neural variability: Friend or foe? Trends Cogn Sci [Internet]. 2015;19(6):322–8. 10.1016/j.tics.2015.04.005. 10.1016/j.tics.2015.04.005 25979849

[34] Anderson J, Rennie C, Gordon E, Howson A, Meares R. Measurement of Maximum Variability within event related potentials in Schizophrenia. Psychiatry Res 39:33–44. 10.1016/0165-1781(91)90006-b 1685249

[35] Shin KS, Kim JS, Kim SN, Hong KS, O’Donnell BF, Chung CK et al. Intraindividual neurophysiological variability in ultra-highrisk for psychosis and schizophrenia patients: Single-trial analysis. NPJ Schizophr [Internet]. 2015;1(1). 10.1038/npjschz.2015.31. 10.1038/npjschz.2015.31 PMC4849455 27336039

[36] Patterson JV, Michalewski HJ, Starr A. Latency variability of the components of auditory event-related potentials to infrequent stimuli in aging, Alzheimer-type dementia, and depression. Electroencephalography and Clinical Neurophysiology/Evoked Potentials Section [Internet]. 1988;71(6):450–60. https://linkinghub.elsevier.com/retrieve/pii/0168559788900494. 10.1016/0168-5597(88)90049-4 2460326

[37] Devos H, Burns JM, Liao K, Ahmadnezhad P, Mahnken JD, Brooks WM (2020). Reliability of P3 event-related potential during working memory across the Spectrum of Cognitive Aging. Front Aging Neurosci.

[38] Hogan MJ, Carolan L, Roche RAP, Dockree PM, Kaiser J, Bunting BP (2006). Electrophysiological and information processing variability predicts memory decrements associated with normal age-related cognitive decline and Alzheimer’s disease (AD). Brain Res.

[39] Kong XZ, Postema MC, Guadalupe T, de Kovel C, Boedhoe PSW, Hoogman M (2022). Mapping brain asymmetry in health and disease through the ENIGMA consortium. Hum Brain Mapp.

[40] Kong XZ, Mathias SR, Guadalupe T, Abé C, Agartz I, Akudjedu TN (2018). Mapping cortical brain asymmetry in 17,141 healthy individuals worldwide via the ENIGMA consortium. Proc Natl Acad Sci U S A.

[41] Jesulola E, Sharpley CF, Bitsika V, Agnew LL, Wilson P. Frontal alpha asymmetry as a pathway to behavioural withdrawal in depression: Research findings and issues. Behavioural Brain Research [Internet]. 2015;292:56–67. 10.1016/j.bbr.2015.05.058. 10.1016/j.bbr.2015.05.058 26051816

[42] Thibodeau R, Jorgensen RS, Kim S. Depression, anxiety, and resting frontal EEG asymmetry: A meta-analytic review. J Abnorm Psychol [Internet]. 2006;115(4):715–29. http://doi.apa.org/getdoi.cfm?doi=10.1037/0021-843X.115.4.715. 10.1037/0021-843X.115.4.715 17100529

[43] Barros C, Pereira AR, Sampaio A, Buján A, Pinal D. Frontal alpha asymmetry and negative Mood: a cross-sectional study in older and younger adults. Symmetry (Basel). 2022;14(8).

[44] Tenke CE, Bruder GE, Towey JP, Leite P, Sidtis JJ. Correspondence between brain ERP and behavioral asymmetries in a dichotic complex tone test [Internet]. 1993. https://psychophysiology.cpmc.columbia.edu/pdf/tenke1993a.pdf. 10.1111/j.1469-8986.1993.tb03205.x 8416063

[45] Ismail Z, Elbayoumi H, Fischer CE, Hogan DB, Millikin CP, Schweizer T et al. Prevalence of Depression in Patients With Mild Cognitive Impairment. JAMA Psychiatry [Internet]. 2017;74(1):58. http://archpsyc.jamanetwork.com/article.aspx?doi=10.1001/jamapsychiatry.2016.3162. 10.1001/jamapsychiatry.2016.3162 27893026

[46] Khatun S, Morshed BI, Bidelman GM. A Single-channel EEG-based approach to detect mild cognitive impairment via speech-evoked brain responses. IEEE Transactions on Neural Systems and Rehabilitation Engineering [Internet]. 2019;27(5):1063–70. https://ieeexplore.ieee.org/document/8693868/. 10.1109/TNSRE.2019.2911970 PMC6554026 30998476

[47] Eyamu J, Kim WS, Kim K, Lee KH, Kim JU. Prefrontal event-related potential markers in association with mild cognitive impairment. Front Aging Neurosci [Internet]. 2023;15(October). https://www.frontiersin.org/articles/10.3389/fnagi.2023.1273008/full. 10.3389/fnagi.2023.1273008 PMC10620700 37927335

[48] Doan DNT, Ku B, Choi J, Oh M, Kim K, Cha W (2021). Predicting Dementia with Prefrontal Electroencephalography and Event-related potential. Front Aging Neurosci.

[49] Choi J, Ku B, Doan DNT, Park J, Cha W, Kim JU et al. Prefrontal EEG slowing, synchronization, and ERP peak latency in association with predementia stages of Alzheimer’s disease. Front Aging Neurosci. 2023;15(March). 10.3389/fnagi.2023.1131857 PMC10076640 37032818

[50] Choi HS, Chung YG, Choi SA, Ahn S, Kim H, Yoon S (2019). Electroencephalographic resting-state functional connectivity of benign epilepsy with centrotemporal spikes. J Clin Neurol (Korea).

[51] Opwonya J, Wang C, Jang KM, Lee K, Kim J, Il, Kim JU. Inhibitory Control of Saccadic Eye Movements and Cognitive Impairment in Mild Cognitive Impairment. Front Aging Neurosci [Internet]. 2022;14:871432. https://www.frontiersin.org/articles/10.3389/fnagi.2022.871432/full. 10.3389/fnagi.2022.871432 PMC9038187 35478701

[52] Petersen RC (2004). Mild cognitive impairment as a diagnostic entity. J Intern Med.

[53] Kang Y, Na DL, Hahn S (1997). A validity study on the Korean MiniMental State Examination (K-MMSE) in dementia patients. J Korean Neurol Association.

[54] Kang Y, Na DL, Hahn S (2003). Seoul Neuropsychological Screening Battery.

[55] Choi J, Ku B, You YG, Jo M, Kwon M, Choi Y et al. Resting-state prefrontal EEG biomarkers in correlation with MMSE scores in elderly individuals. Sci Rep [Internet]. 2019;9(1):1–15. 10.1038/s41598-019-46789-2. 10.1038/s41598-019-46789-2 PMC6639387 31320666

[56] Anderson J, Gordon E, Barry RJ, Rennie C, Gonsalvez C, Pettigrew G (1995). Event related response variability in schizophrenia: effect of intratrial target subsets. Psychiatry Res.

[57] n der Vinne N, Vollebregt MA, van Putten MJAM, Arns M (2017). Frontal alpha asymmetry as a diagnostic marker in depression: fact or fiction? A meta-analysis. Neuroimage Clin.

[58] Wei T, Simko V. R package corrplot: Visualization of a Correlation Matrix [Internet]. 2021. https://github.com/taiyun/corrplot.

[59] R Core Team. R: A Language and Environment for Statistical Computing [Internet]. Vienna, Austria: R Foundation for Statistical Computing. 2022. https://www.r-project.org/.

[60] Wickham H. ggplot2: Elegant Graphics for Data Analysis [Internet]. Springer-Verlag New York. 2016. https://ggplot2.tidyverse.org.

[61] Sjoberg, Daniel D, Whiting K, Curry M, Lavery, Jessica A, Larmarange J. Reproducible Summary Tables with the gtsummary Package [Internet]. Vol. 13, The R Journal. 2021. p. 570. 10.32614/RJ-2021-053.

[62] Kim S, Lee G, Yoo H. Effect of aging and physical activity on cognitive function: an examination of P300. International Journal of Digital [Internet]. 2013;24:597–606. http://search.proquest.com/openview/9710e8be0186259142dd5f4b6443ca54/1?pq-origsite=gscholar.

[63] Vecchio F, Määttä S. The Use of Auditory Event-Related Potentials in Alzheimer’s Disease Diagnosis. Int J Alzheimers Dis [Internet]. 2011;2011:1–7. http://www.hindawi.com/journals/ijad/2011/653173/. 10.4061/2011/653173 PMC3100636 21629759

[64] Picton TW, Hillyard SA. Human auditory evoked potentials. II: Effects of attention. Electroencephalogr Clin Neurophysiol [Internet]. 1974;36:191–200. https://linkinghub.elsevier.com/retrieve/pii/0013469474901564. 10.1016/0013-4694(74)90156-4 4129631

[65] Delano-Wood L, Bondi MW, Sacco J, Abbeles N, Jak JA, Libon JD et al. Heterogeneity in mild cognitive impairment: Differences in neuropsychological profile and associated white matter lesion pathology. Journal of the International Neuropsychological Society [Internet]. 2009;15(6). https://www.ncbi.nlm.nih.gov/pmc/articles/PMC3624763/pdf/nihms412728.pdf. 10.1017/S1355617709990257 PMC3034688 19891820

[66] Tenke CE, Kayser J, Fong R, Leite P, Towey JP, Bruder GE. Response-and Stimulus-Related ERP Asymmetries in a Tonal Oddball Task: A Laplacian Analysis. Vol. 10, Brain Topography. 1998. 10.1023/a:1022261226370 9562541

[67] Cecchi M, Moore DK, Sadowsky CH, Solomon PR, Doraiswamy PM, Smith CD (2015). A clinical trial to validate event-related potential markers of Alzheimer’s disease in outpatient settings. Alzheimer’s Dementia: Diagnosis Assess Disease Monit.

[68] Chapman RM, Nowlis GH, McCrary JW, Chapman JA, Sandoval TC, Guillily MD (2007). Brain event-related potentials: diagnosing early-stage Alzheimer’s disease. Neurobiol Aging.

[69] Stuckenschneider T, Askew CD, Weber J, Abeln V, Rüdiger S, Summers MJ et al. Auditory event-related potentials in individuals with subjective and mild cognitive impairment. Behavioural Brain Research [Internet]. 2020;391(October 2019):112700. 10.1016/j.bbr.2020.112700. 10.1016/j.bbr.2020.112700 32446915

[70] Chapman RM, McCrary JW, Gardner MN, Sandoval TC, Guillily MD, Reilly LA et al. Brain ERP components predict which individuals progress to Alzheimer’s disease and which do not. Neurobiol Aging [Internet]. 2011;32(10):1742–55. 10.1016/j.neurobiolaging.2009.11.010. 10.1016/j.neurobiolaging.2009.11.010 PMC2902777 20005599

[71] Ganapathi AS, Glatt RM, Bookheimer TH, Popa ES, Ingemanson ML, Richards CJ (2022). Differentiation of subjective cognitive decline, mild cognitive impairment, and Dementia using qEEG/ERP-Based cognitive testing and volumetric MRI in an Outpatient Specialty Memory Clinic. J Alzheimer’s Disease.

