# Supplementary material for: Prefrontal intra-individual ERP variability and its asymmetry: exploring its biomarker potential in mild cognitive impairment
Source: Alzheimers Res Ther. 2024 Apr 13;16:83. doi: 10.1186/s13195-024-01452-5 (PMC11015694; doi:10.1186/s13195-024-01452-5)
Supplement: Supplementary file 1 — Supplementary Material 1 [file 13195_2024_1452_MOESM1_ESM.pdf]

Prefrontal Intra-individual ERP Variability and its Asymmetry: Exploring its Biomarker Potential in Mild Cognitive Impairment.

Joel Eyamu[1,2], Wuon-Shik Kim[1], Kahye Kim[1], Kun Ho Lee[3,4,5] and Jaeuk U. Kim [1,2*]

[1]Digital Health Research Division, Korea Institute of Oriental Medicine, Daejeon, South Korea
[2]KM Convergence Science, University of Science and Technology, Daejeon, South Korea

[3]Gwangju Alzheimer's Disease and Related Dementias (GARD) Cohort Research Center, Chosun University, Gwangju, South Korea

[4]Department of Biomedical Science, Chosun University, Gwangju, South Korea
[5]Dementia Research Group, Korea Brain Research Institute, Daegu, South Korea

Figure **S1:** Composition of the participants in the study.

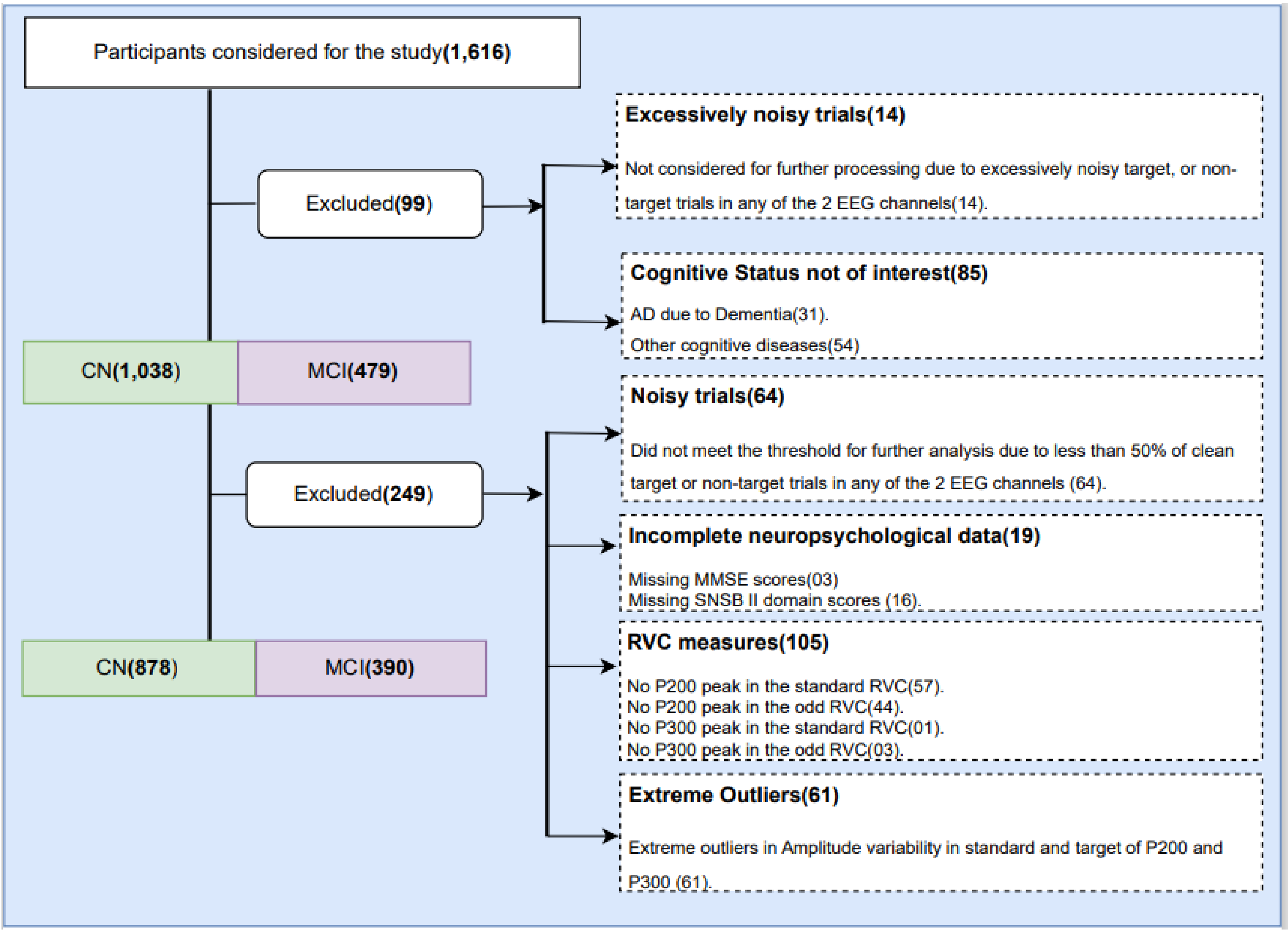

Figure S1: Summary of the composition of the participants in the study; CN: Cognitively normal; MCI: Mild cognitive impairment; AD: Alzheimer's disease; RVC: Response variance curve; Extreme outliers are values above Q3 + 3xIQR or below Q1 - 3xIQR, where Q1 and Q3 are the first and third quartile, respectively and IQR is the interquartile range (IQR = Q3 - Q1); All values are in parentheses.

Table S1: Detailed description of the variables considered in the study

Variable	Unit	Description
**RVC measures**		
AMPV	μV^2	**Amplitude Variability**: The largest variance point (peak) in the measurement window is surrounded by lower values on both sides of the RVC curve. The consolidated value is calculated by getting the average of the variability of the left and right hemispheres as; AMPV = (AMPV_r + AMPV_l)/ 2; *Where* AMPV_r and AMPV_l is the AMPV value of the right and left hemispheres respectively
AUCV	μV^2ms	**AUC Variability**: The sum of the amplitude variance in a given time window after the auditory stimulus onset. The consolidated value for analysis is calculated by getting the average of the variability of the left and right hemispheres as; AUCV = (AUCV_r + AUCV_l)/ 2; *Where* AUCV_r and AUCV_l is the AUCV value of the right and left hemispheres respectively.
LATV	ms	**Latency Variability**: The timepoint corresponding to the peak variance of the amplitude starting from the stimulus onset.
FALV	ms	**Fractional area Latency variability:** The timepoint in which the AUCV is divided into two equal parts.
**RVC Asymmetry measures**		
AMPV_Asym	-	**Amplitude Variability Asymmetry**<br>AMPV_Asym = (AMPV_r – AMPV_l)/ (AMPV_l +AMPV_r)<br>*Where* AMPV_r and AMPV_l are AMPV values of the right and left hemispheres respectively
AUCV_Asym	-	**AUC Variability Asymmetry**<br>AUCV_Asym = (AUCV_r – AUCV_l)/ (AUCV_l +AUCV_r)<br>*Where* AUCV_r and AUCV_l are the AUCV values of the right and left hemispheres respectively
LATV_Asym	-	**LAT Variability Asymmetry**<br>LATV_Asym = (LATV_r – LATV_l)/ (LATV_l +LATV_r)<br>*Where* LATV_r and LATV_l are the LATV values of the right and left hemispheres respectively.
FALV_Asym	-	**FAL Variability Asymmetry**<br>FALV_Asym = (FALV_r – FALV_l)/ (FALV_l +FALV_r)<br>*Where* FALV_r and FALV_l is the FALV value of the right and left hemispheres respectively

Table S2: Differences in ERP, RVC, and behavioral measures in the two groups

Characteristic	CN, N = 878[1]	MCI, N = 390[1]	T- statistic	p-value[2]
**P3 RVC asymmetry**				
**AMPV_Asym**	0.10 (0.23)	0.14 (0.23)	-2.32	**0.021**
**LATV_Asym**	-0.03 (0.13)	-0.02 (0.13)	-0.98	0.3
**AUCV_Asym**	0.02 (0.21)	0.05 (0.21)	-2.24	**0.025**
**FALV_Asym**	-0.01 (0.03)	0.00 (0.03)	-1.62	0.11
**P2 Standard variability**				
**AMPV**	124.28 (76.02)	140.47 (88.20)	-3.14	**0.002**
**LATV**	231.92 (30.10)	232.06 (31.09)	-0.08	>0.9
**FALV**	229.55 (6.37)	229.97 (6.82)	-1.08	0.3
**AUCV**	3,922.47 (2,379.03)	4,430.38 (2,845.23)	-3.08	**0.002**
**P2 Target variability**				
**AMPV**	113.50 (74.94)	125.80 (83.34)	-2.60	**0.009**
**LATV**	231.39 (29.62)	235.57 (28.63)	-2.35	**0.019**
**FALV**	229.80 (6.64)	230.53 (6.76)	-1.80	0.071
**AUCV**	3,570.20 (2,326.34)	3,951.85 (2,564.76)	-2.61	**0.009**
**P3 Standard variability**				
**AMPV**	183.59 (123.83)	205.66 (140.22)	-2.68	**0.008**
**LATV**	443.40 (64.09)	449.46 (66.50)	-1.54	0.12
**FALV**	448.41 (15.72)	449.41 (17.04)	-1.01	0.3
**AUCV**	10,119.64 (6,784.98)	11,296.71 (7,707.85)	-2.60	**0.009**
**P3 Target variability**				
**AMPV**	172.06 (126.85)	192.05 (133.35)	-2.55	**0.011**
**LATV**	445.13 (64.62)	449.33 (67.28)	-1.05	0.3
**FALV**	448.38 (16.61)	449.46 (17.75)	-1.05	0.3
**AUCV**	9,409.95 (6,982.16)	10,425.44 (7,245.65)	-2.36	**0.018**

[1]Mean (*SD*); [2]Two Sample t-test; Pearson's Chi-squared test; Welch Two Sample t-test; Significant variables (*p*-value ≤ 0.05) are bolded

Table S3. Estimated OR and 95% CI for ERP, RVC & behavioral measures derived from LR models

	Model 1			Model 2			Model 3		
**Variables**	**OR[1]**	**95% CI[1]**	**p-value**	**OR[1]**	**95% CI[1]**	**p-value**	**OR[1]**	**95% CI[1]**	**p-value**
**P3 variability Asymmetry**									
**AMPV_Asym**	1.15	1.02, 1.30	**0.021**	1.15	1.02, 1.30	**0.021**	1.16	1.02, 1.32	**0.020**
**LATV_Asym**	1.06	0.94, 1.20	0.33	1.07	0.95, 1.21	0.28	1.05	0.92, 1.19	0.46
**AUCV_Asym**	1.15	1.02, 1.29	**0.025**	1.15	1.02, 1.29	**0.024**	1.16	1.02, 1.31	**0.024**
**FALV_Asym**	1.10	0.98, 1.24	0.11	1.11	0.98, 1.25	0.10	1.09	0.96, 1.24	0.17
**P2 Standard variability**									
**AMPV**	1.22	1.08, 1.36	**0.001**	1.22	1.09, 1.38	**<0.001**	1.20	1.06, 1.36	**0.004**
**LATV**	1.00	0.89, 1.13	0.94	1.00	0.89, 1.13	>0.99	1.02	0.90, 1.16	0.78
**FALV**	1.07	0.95, 1.20	0.28	1.07	0.94, 1.20	0.30	1.07	0.94, 1.22	0.30
**AUCV**	1.21	1.08, 1.36	**0.001**	1.23	1.09, 1.38	**<0.001**	1.20	1.06, 1.36	**0.005**
**P2 Target variability**									
**AMPV**	1.17	1.04, 1.31	**0.010**	1.19	1.05, 1.33	**0.005**	1.18	1.04, 1.33	**0.011**
**LATV**	1.15	1.02, 1.30	**0.019**	1.16	1.02, 1.31	**0.019**	1.15	1.01, 1.31	**0.033**
**FALV**	1.11	0.99, 1.25	0.072	1.11	0.98, 1.25	0.094	1.10	0.97, 1.25	0.13
**AUCV**	1.17	1.04, 1.31	**0.010**	1.19	1.06, 1.34	**0.004**	1.18	1.04, 1.33	**0.012**
**P3 Standard variability**									
**AMPV**	1.18	1.05, 1.32	**0.006**	1.18	1.05, 1.32	**0.007**	1.16	1.03, 1.32	**0.017**
**LATV**	1.10	0.97, 1.24	0.12	1.10	0.97, 1.24	0.13	1.11	0.97, 1.26	0.12
**FALV**	1.06	0.94, 1.20	0.31	1.06	0.94, 1.20	0.33	1.05	0.93, 1.20	0.42
**AUCV**	1.17	1.04, 1.32	**0.007**	1.18	1.05, 1.32	**0.007**	1.16	1.02, 1.31	**0.022**
**P3 Target variability**									
**AMPV**	1.16	1.03, 1.30	**0.012**	1.17	1.04, 1.32	**0.008**	1.18	1.04, 1.33	**0.011**
**LATV**	1.07	0.95, 1.20	0.29	1.08	0.95, 1.22	0.23	1.10	0.97, 1.25	0.14
**FALV**	1.07	0.95, 1.20	0.29	1.07	0.95, 1.20	0.30	1.07	0.95, 1.22	0.28
**AUCV**	1.15	1.02, 1.29	**0.020**	1.17	1.04, 1.31	**0.011**	1.16	1.03, 1.32	**0.018**

Table S2: [1]OR = Odds ratio, [2]CI = Confidence interval, LR = Logistic regression; **Model 1**: The unadjusted LR model; **Model 2**: LR model adjusted for demographic characteristics of age, sex, and years of education; **Model 3**: LR model adjusted for demographic characteristics and the MMSE score. Significant variables (*p*-value ≤ 0.05) are bolded.
